# Supplementary material for: Explore the shared molecular mechanism between dermatomyositis and nasopharyngeal cancer by bioinformatic analysis
Source: PLoS One. 2024 May 16;19(5):e0296034. doi: 10.1371/journal.pone.0296034 (PMC11098312; doi:10.1371/journal.pone.0296034)
Supplement: S1 File — (DOCX) [file pone.0296034.s001.docx]

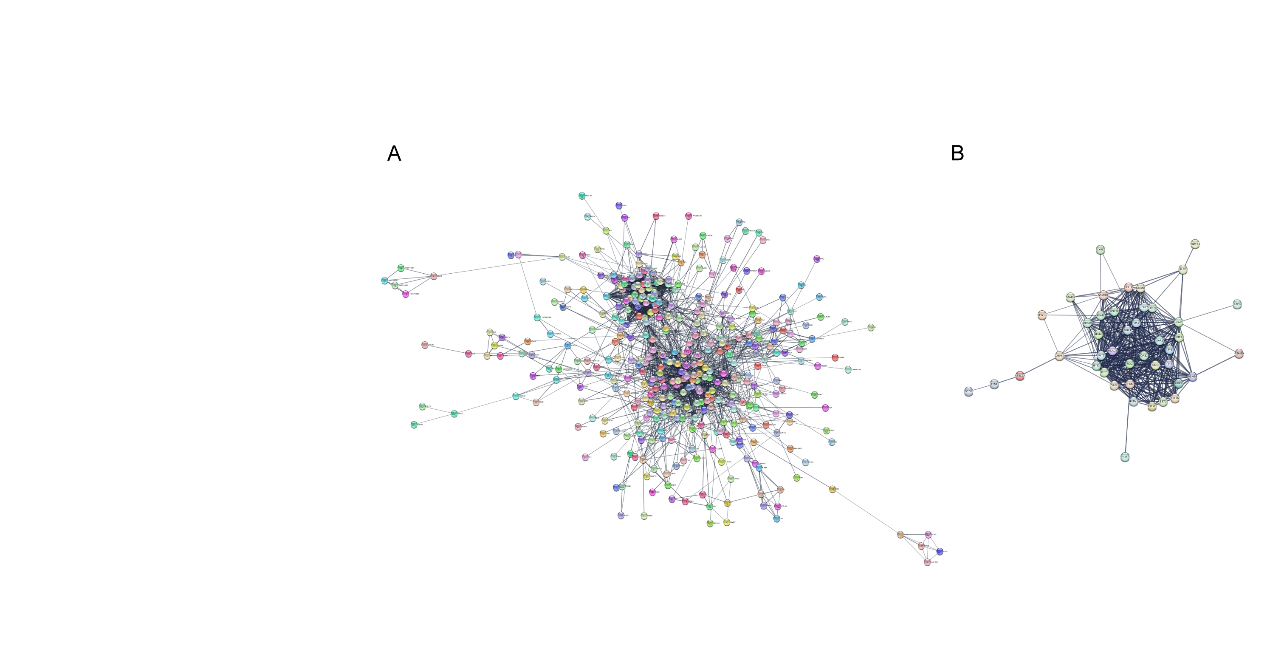


**S1 Fig. Protein–protein interaction network (PPI)** (A) PPI of genes in the red module of DM. (B) PPI of genes in the purple module of NPC.


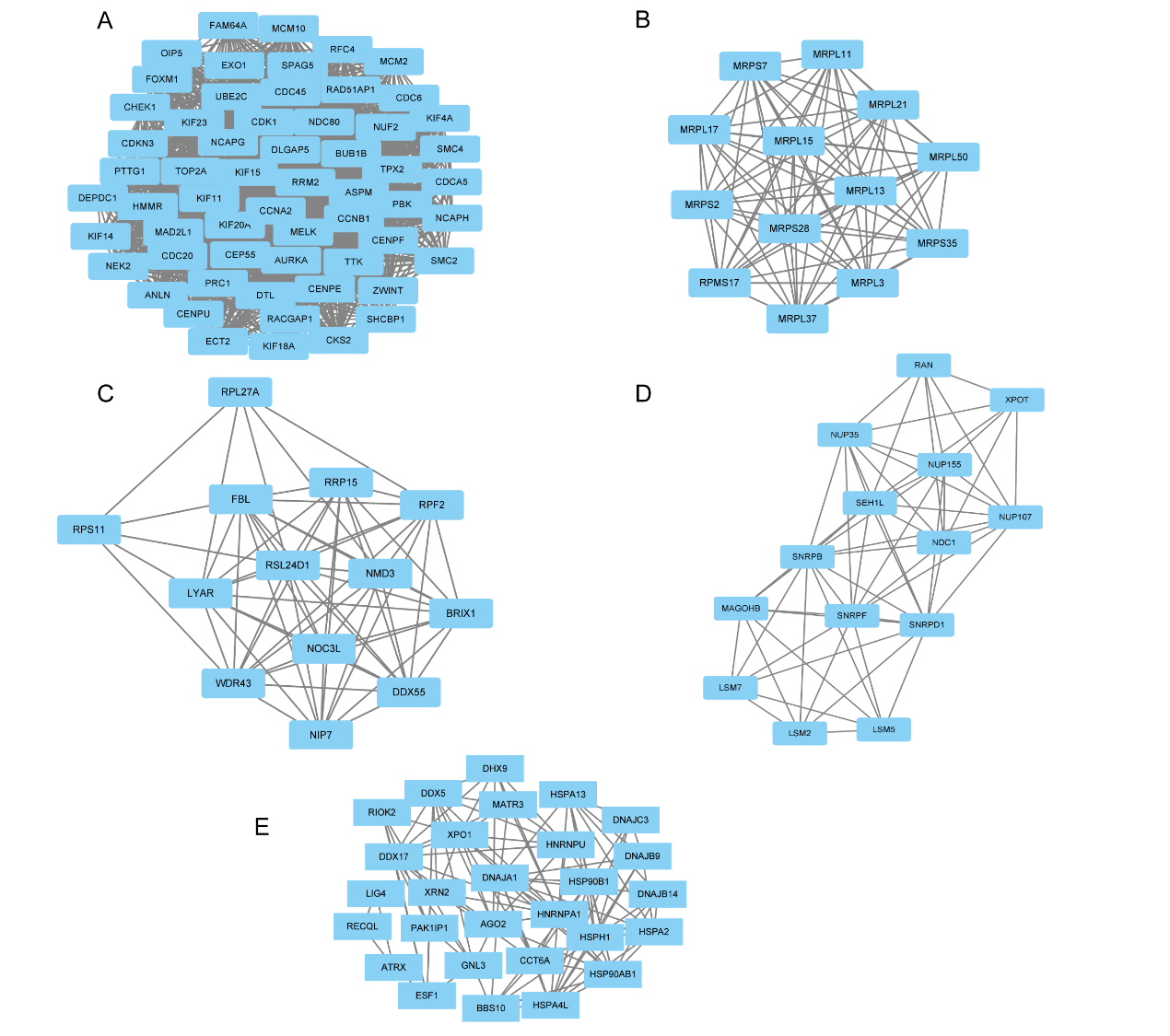


**S2 Fig. The PPI network and clusters analysis.** (A-D) The subnetwork 1 to 4 were withdrawn from the blue module of NPC. (E) The subnetwork has withdrawn from the brown module of NPC.


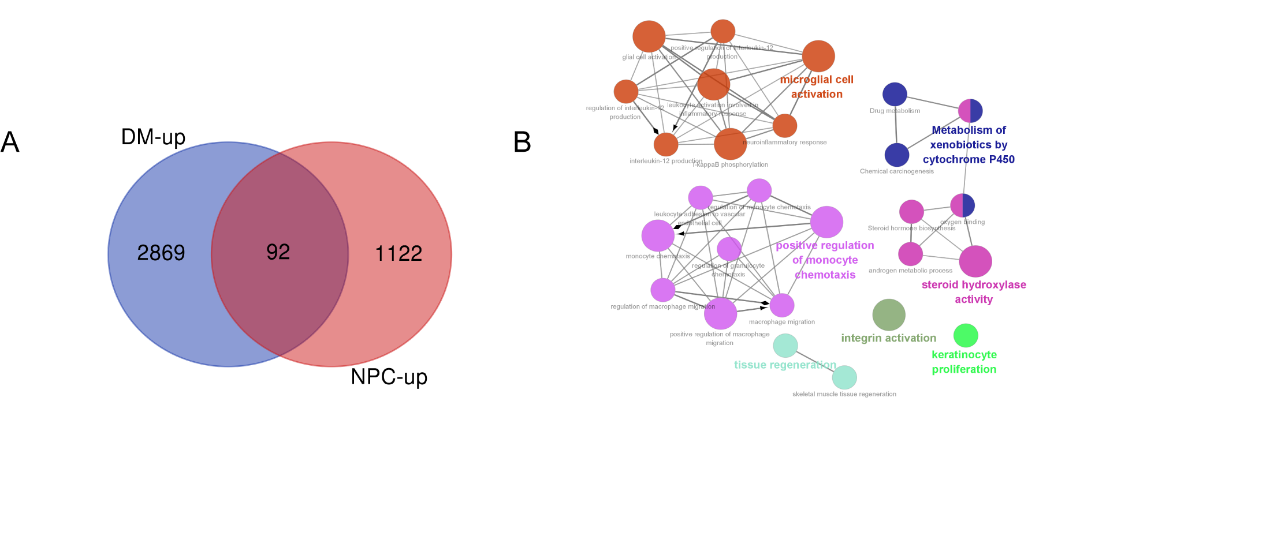


**S3 Fig. Common up-regulated genes and enrichment analysis by ClueGO in DM and NPC.** (A) The Venn diagram showed 92 common up-regulated genes overlapped in GSE142807 and GSE53819. (B) The GO and KEGG analysis of the common up-regulated DEGs.
