# Supplementary material for: Explore the shared molecular mechanism between dermatomyositis and nasopharyngeal cancer by bioinformatic analysis
Source: PLoS One. 2024 May 16;19(5):e0296034. doi: 10.1371/journal.pone.0296034 (PMC11098312; doi:10.1371/journal.pone.0296034)
Supplement: S1 Table — (DOCX) [file pone.0296034.s002.docx]

**S1 Table. Generalize of four datasets in DM and NPC**
