# Supplementary material for: Explore the shared molecular mechanism between dermatomyositis and nasopharyngeal cancer by bioinformatic analysis
Source: PLoS One. 2024 May 16;19(5):e0296034. doi: 10.1371/journal.pone.0296034 (PMC11098312; doi:10.1371/journal.pone.0296034)
Supplement: Attachment — Submitted filename: Plos One -- PONE-D-23-24175 Review.docx [file pone.0296034.s010.docx]

**Explore the shared molecular mechanism between dermatomyositis and nasopharyngeal cancer by bioinformatic analysis**- **PONE-D-23-24175**

The following questions should be addressed by the author for further processing of the manuscript

1. **Line 45 and 46**: Could you elaborate on the specific genetic, immunological, and environmental factors mentioned as potential contributors to DM development?
2. **Line 52:** The line mentions that type I interferon signaling and myositis antibodies have a significant role in DM development. Could you provide more insight into how these factors contribute to the pathophysiology of DM?
3. **Line 55 and 56:** The passage suggests a high risk of DM patients developing nasopharyngeal carcinomas (NPC). What mechanisms or pathways could potentially link DM and NPC?
4. **Line 61**: The passage mentions a poorer prognosis for patients with NPC and DM due to stronger drug resistance and more severe radiotherapy complications. Could you elaborate on the reasons behind these observations?
5. **Line 80:** Could you elaborate on the process of identifying gene sets associated with DM and NPC phenotypes using the WGCNA package? How was the threshold for gene expression variation determined (standard deviation≤0.5)?
6. **Line 84**: What are the underlying reasons for choosing the specific soft power value of 9 for WGCNA analysis?
7. **Line 89:** How does the hierarchical clustering of gene trees based on dissimilarity help identify distinct gene modules? Could you explain the significance of the parameters "minModuleSize=50", "deepSplit=2", and "cutHeight=0.25" in grouping gene modules?
8. **Line 95:** What insights did the Venn analysis provide in terms of shared genes between DM and NPC? How were these shared genes further analyzed using Gene Ontology (GO) and Kyoto Encyclopedia analyses (KEGG)?
9. **Line 100:** Can you provide more details about the protein-protein interaction (PPI) networks constructed using the STRING tool? How were the parameters "degree cutoff=6", "node score cutoff=0.2", and "K-core=6" determined for the MCODE algorithm?
10. **Line 107:** How did you decide on the cutoff values "∣logFC∣=1" and "adjusted p-values<0.05" for categorizing genes as up-regulated or down-regulated?
11. **Line 108:** What were the key biological processes identified by ClueGO based on common DEGs between DM and NPC?
12. **Line 125:** Could you provide more details about CIBERSORT and CIBERSORT-R package? How were these tools used to predict immune cell infiltration levels within pathological tissues? Can you add the reference?
13. **Line 133**: How did you assess the ability of pivotal genes to predict disease using pROC analysis? What were the specific criteria for categorizing genes into two expression level groups?
14. **Line 148**: Could you provide more context on the significance of the co-expression modules that showed significant positive correlations with DM and NPC? How do these modules reflect the underlying molecular mechanisms associated with the conditions?
15. **Line 157:** How were the shared genes between DM and NPC identified and validated? What were the specific criteria for selecting these shared genes?
16. **Line 161:** Could you elaborate on how the Gene Ontology (GO) analysis and Kyoto Encyclopedia analyses (KEGG) provided insights into the biological processes and pathways related to viral infections and immune responses?
17. **Line 166**: How were the protein-protein interaction (PPI) networks constructed for the key modules of DM and NPC? What criteria were used to cluster the PPI network using the MCODE algorithm?
18. **Line 171:** Can you explain the biological significance of the shared pathways, such as negatively regulating viral genome replication and response to type I interferon, as visualized by ClueGO?
19. **Line 179** : What was the rationale for selecting specific cutoff values for categorizing DEGs as up-regulated or down-regulated?
20. **Line 205 and 206**: How do the correlations between immune cell infiltration and hub genes help uncover potential immune mechanisms underlying DM and NPC?
21. **Line 210 and 211:** How were the diagnostic performance and predictive capabilities of hub genes evaluated using the pROC package?
22. **Line 219:** What insights were gained from the Gene Set Enrichment Analysis (GSEA) regarding the role of hub genes in macrophage-related processes and viral regulation?
23. **Line 235**: Could you explain in more detail how viral infections, such as Coxsackievirus RNA and Coronavirus-19, can trigger or mimic autoimmune diseases like DM? How do these infections influence the immune response and contribute to the pathophysiology of DM?
24. **Line 263**: Can you elaborate on the potential shared pathophysiology between DM and malignancies, particularly nasopharyngeal carcinoma (NPC)? How might the identified hub genes (IFI44L, IFIT1, IFI6, GBP1, IFX1, OAS1, IFI44, ISG15, IFIT3, and BST2) be involved in this shared pathophysiology?
25. **Line 274:** How does miR-146a function as a key immunoregulatory factor in autoimmune diseases like SLE and DM? How does its regulation by type I interferons and Toll-like receptors contribute to its role in these diseases?
26. **Line 297:** Could you provide more insight into how the identified hub genes (IFI44L, IFIT1, IFI6, GBP1, IFX1, OAS1, IFI44, ISG15, IFIT3, and BST2) influence viral infection, immune responses, and potentially the development of malignancy? How do these genes interact with each other and with other cellular processes?
