# Supplementary material for: Explore the shared molecular mechanism between dermatomyositis and nasopharyngeal cancer by bioinformatic analysis
Source: PLoS One. 2024 May 16;19(5):e0296034. doi: 10.1371/journal.pone.0296034 (PMC11098312; doi:10.1371/journal.pone.0296034)
Supplement: Attachment — Submitted filename: Response to Reviewers.docx [file pone.0296034.s011.docx]

**Explore the shared molecular mechanism between dermatomyositis and nasopharyngeal cancer by bioinformatic analysis**- **PONE-D-23-24175**

Dear reviewers:

Thank you for your decision and constructive comments on my manuscript. We have carefully considered the suggestion of Reviewer and make some changes. We have tried our best to improve and made some changes in the manuscript. According to your comments. Revision notes, point-to-point, are given as follows:

1. **Line 45 and 46**: Could you elaborate on the specific genetic, immunological, and environmental factors mentioned as potential contributors to DM development?

**Response:**

Thank you for your valuable feedback. Inflammatory myopathies in different regions identify unique risk HLA haplotypes. For example, HLA-DQA1*0601 is identified in DM patients in the U.S., whereas HLA-DRB1*07 is involved in DM patients in China. Risk HLA haplotypes correlate strongly with antibodies against inflammatory myopathies. HLA-DRB1 * 07 is associated with anti-Mi-2 autoantibodies, which have a prevalence of 2 - 50 % in DM and are associated with typical DM lesions. Haplotype HLA-DRB1 * 03, HLA-DQA1 * 05 and HLA-DQB1 * 02 are risk factors for anti-aminoacyl- t RNA synthetase (ARS) autoantibodies. In addition, non-HLA susceptibility genes such as *BLK* and *STAT4* in Japanese, *IRF5* in Chinese were also identified^[1]^. Single nucleotide polymorphisms in the *PTPN22* gene are associated with juvenile dermatomyositis, and the complex phosphatase encoded by the *PTPN22* gene may selectively promote the production of type I interferon from bone marrow-derived macrophages by enhancing the pattern recognition receptor activated(TCR)^[2]^.Epigenetics is also involved in the pathogenesis of myositis, with more than 20 genes with significant methylation differences between juvenile DM and normal controls, and DNA methylation modifications may have a regulatory role for about 20% of the differentially expressed genes. miRNAs are also increasingly being investigated in DM/PM, with miR -1, miR - 133a, and miR - 133b down-regulated and miR - 146a, miR-221 and miR - 222 were overexpressed. All of these miRNAs are involved in myoblast differentiation and muscle weakness. miR - 146a also negatively regulates the type 1 IFN pathway^[3]^.

In a twin cohort study, significant differences in myositis autoantibody profiles were found between twins. The marked difference between the autoantibody levels of seropositive patients and the corresponding subgroup of unaffected twins supports the piont that autoantibody production is not controlled by genetic risk factors alone, but requires the interaction of environmental factors and genetics to influence the autoimmune response^[4]^. A variety of environmental factors may activate the DM disease autoimmune response. Viral infections such as coxsackie, minuscule viruses, and influenza viruses are often associated with childhood myositis. Human myxovirus resistance A (Mx A) is an interferon α / β-inducible protein that interferes with viral assembly and provides innate defense against several ribonucleic acid (RNA) viruses. MxA has a perifascicular distribution in DM patients and selectively expressed in endothelial cells, which is present in the early stages of the disease^[5]^. Uruha and collogues et al. found that the expression of sarcoplasmic MxA was more reliable for the diagnosis of DM than perifascicular atrophy^5^. Ultraviolet light exposure promotes the production of anti-Mi2 antibodies and induces the appearance of skin damage in DM^[6]^. Sun exposure and the use of NSAIDs are important environmental risk factors for the recurrence of dermatomyositis^[7]^.

2. **Line 52:** The line mentions that type I interferon signalling and myositis antibodies have a significant role in DM development. Could you provide more insight into how these factors contribute to the pathophysiology of DM?

**Response:**

Thank you for your questions. Type I IFN are mainly produced by plasmacytoid dendritic cells in physiologic state. The type I interferon response is predominant in DM and is particularly enhanced in anti-melanoma differentiation-related gene 5 (MDA5) + DM. In vivo and in vitro transcriptomic studies of blood and target tissues (e.g., skin and muscle) from patients with IIM emphasize the critical role of IFN in inducing and maintaining disease manifestations. Abnormalities in mitochondrial morphology and superoxide regulation in JDM CD14+ monocytes lead to increased oxidized mitochondrial DNA, and the dysregulation of mitochondrial DNA is associated with upregulation of type I IFN in JDM^[8]^. LL - 37 may activate type I IFNs, which initiate and maintain the inflammatory process. Prolonged exposure of the immune system to type I IFNs may eventually break immune tolerance and lead to autoimmune myositis^[9]^.In subjects with a background genetic susceptibility to dermatomyositis, activation of MDA5 during viral infection may lead to overexpression of type I IFN by pDCs, resulting in enhanced antigen presentation by antigen-presenting cells (APCs) and antibody production by plasma cells. Type I IFN stimulates the maturation of B cells into plasma cells, which in turn produces anti-MDA5 autoantibodies. Anti-MDA5 autoantibodies are involved in the formation of immune complexes (ICs), which promote IFN production by stimulating Toll-like receptor 7 (TLR7). The interaction between anti-MDA5 autoantibodies and type I IFN creates a vicious circle. Anti-MDA5 autoantibodies also contribute to the maintenance of the inflammatory response and endothelial damage through the release of neutrophil extracellular trapping networks (NETS) from activated neutrophils^[10]^.

Gallay and colleagues found that overproduction of type I IFN leads to impaired proliferation of muscle stem cells in DM patients, leading to defects in muscle repair. In addition, type I IFN can lead to myotube mitochondrial damage by increasing reactive oxygen species production, which is one of the nonimmune mechanisms of IIM muscle damage^[11]^.

3. **Line 55 and 56:** The passage suggests a high risk of DM patients developing nasopharyngeal carcinomas (NPC). What mechanisms or pathways could potentially link DM and NPC?

**Response:**

Thank you very much for your comments and professional advice. Study shown that Epstein-Barr virus (EBV) anti-viral capsid antigen IgA antibody (VCA - IgA) were higher in NPC patients with DM than in the NPC group without DM^[12]^. Additionally, EBV genome positive rate was significantly higher in patients with DM combined with NPC than in patients without NPC. Suggesting a positive correlation between EBV virus infection and DM complicating NPC^[13]^. The type I interferon (IFN-α / β) system plays a very important role in controlling viral infection by promoting the synthesis of various antiviral proteins such as ISG15, and is also an important pathogenesis of dermatomyositis. However, the EBV oncogene LMP1 can promote viral infection and tumorigenesis by degrading RIG-I, inhibiting virus-mediated type I interferon production and down-regulating the RIG-I signaling pathway^[14]^. The programmed death-1 (PD-1) checkpoint pathway can achieve tolerance to self-antigens by down-regulating the immune response. It was found that approximately 70% of EBV-positive NPC patients express PD-L1^[15]^. And higher serum levels of soluble PD-L1 in patients with DM associated with malignancy than in patients without malignancy^[16]^. Which may provide evidence that EBV promotes PD-L production by NPC tumour cells and attenuates local and systemic immune responses. In addition, sustained exposure to antigens in myositis may lead to T-cell depletion and senescence, resulting in fewer effective T cells invading skeletal muscle, and this reduced degree of inflammation in myofibrillar tissues is thought to be associated with PD-1/PD-L1^[17]^. In this study, we concluded that down-regulation of antiviral-related genes and reduced type I IFN interferon response may be an important pathogenetic mechanism in DM complicated with nasopharyngeal carcinoma. Previous studies have shown that low serum CPK levels in DM patients with concomitant malignancy represent a low degree of myositis inflammation, supporting the conclusion that the immunoinflammatory response is reduced in DM patients with concomitant malignancy^[18]^.

4. **Line 61**: The passage mentions a poorer prognosis for patients with NPC and DM due to stronger drug resistance and more severe radiotherapy complications. Could you elaborate on the reasons behind these observations?

**Response**:

Thank you for your question. Clinical cohort studies have shown that patients with dermatomyositis with nasopharyngeal carcinoma have a significantly lower survival rate and poor prognosis^[19]^ and severe radiotherapy complications^[20]^. Tumour radiotherapy enhances the anti-tumour immune response of CD8+ T-cells by radiation-induced activation of STING signalling and subsequent type I interferon (IFN-I) production^[21]^. Excessive type I IFN production leads to skin and muscle damage, thereby exacerbating dermatomyositis symptoms. Therefore, radiation-induced IFN-I overactivation may be one of the reasons for the severe complications of dermatomyositis associated with radiotherapy for nasopharyngeal carcinoma. Type I IFN is not only an important proinflammatory factor in dermatomyositis, but also crucial for inducing the efficacy of chemotherapy and radiotherapy. Studies have shown that the efficacy of chemotherapy is determined by T cell-associated immunity. IFN - I signalling is essential for specific anti-tumour T cell responses. Various chemotherapeutic agents such as cisplatin or STING agonists induce IFN-I production to exert anti-tumour effects. However, tumour cells may inhibit STING levels and IFN-I production by increasing the expression of tryptophan metabolites or high expression of the aromatic hydrocarbon receptor AhR, enhancing patient treatment resistance^[22]^. In addition, tumour cell-intrinsic triple structural domain protein 21 (TRIM21) also inhibits radiation-induced type-I IFN signalling-mediated anti-tumour immune responses, thereby increasing resistance to radiotherapy and affecting efficacy and prognosis^[23]^. All the above studies indicate that type I IFN signalling pathway mediate the pathogenesis of nasopharyngeal carcinoma complicated by dermatomyositis, and even be involved in drug resistance and affect the prognosis.

5. **Line 80:** Could you elaborate on the process of identifying gene sets associated with DM and NPC phenotypes using the WGCNA package? How was the threshold for gene expression variation determined (standard deviation≤0.5)?

**Response**:

Thank you for your questions. **Line 95**, we added the references. Through the WCGNA weighted analysis, the most highly correlated genes are filtered out by comparing with the healthy control group. The main steps are as follows: 1. Determine the soft threshold and construct the adjacency matrix; 2. Convert the adjacency matrix into a topological overlap matrix to reduce false correlations; 3. Identify highly correlated gene modules, where genes within the same module have similar biological functions; 4. Identify the key models by association analysis of gene modules with disease phenotypes; 5. Functional enrichment analysis, where GO and KEGG enrichment analysis was performed on genes within the respective key gene modules of DM and NPC; 6. Functional enrichment analysis, GO and KEGG enrichment analysis of the internal genes in the key gene modules of DM and NPC; 7. Identification of hub genes, intersection of the key gene modules of the two diseases enriched with the same functional pathway, and obtaining genes that are significantly related to both DM and NPC.

In the gene matrix, when a gene has a small expression variance in the vast majority of samples, the gene needs to be filtered by the expression mean or expression variance of that gene. When the expression level of the gene fluctuates little in the majority of samples (sd ± 0.5), it will be filtered out.

6. **Line 84**: What are the underlying reasons for choosing the specific soft power value of 9 for WGCNA analysis?

**Response**:

We thank the reviewer for raising this question. The soft threshold β in statistics is an index to test the validity of research work, the larger the value of β, the more reliable the results of the study. β value in WGCNA is the value of correlation between genes and whether it conforms to the scale-free network. In order to comply with the scale-free network, the smallest β value when the scale-free fit index (signed R^2) > 0.8 or when the plateau period is gradually reached is generally recognized as the best power for constructing the scale-free network. According to the above criteria, the GSE46239 dataset applies a β value of 9 and the dataset GES12452 has a β value of 5(**Line 99-Line 100)**.

7. **Line 89:** How does the hierarchical clustering of gene trees based on dissimilarity help identify distinct gene modules? Could you explain the significance of the parameters "minModuleSize=50", "deepSplit=2", and "cutHeight=0.25" in grouping gene modules?

**Response:**

Thank you for your questions. In WGCNA analysis, in order to comply with the scale-free topology criterion, a neighbor-joining matrix (Adjacency matrix) is formed by the weighted correlation values between genes and genes, which is then converted into a topological overlap matrix (TOM) based on the expression weight values of individual genes in order to reduce the pseudocorrelation. Next, genes with similar expression patterns are clustered into the same module, while gene correlations between modules are relatively low. The expression of a gene was correlated with the module eigengene, and the higher the correlation coefficient (Module Membership, MM), the higher the correlation between the gene and the module.

"MinModuleSize=50" means that the minimum number of genes per module is 50. "deepSplit" is the cutting strength, the larger the value, the more sensitive and the more modules are obtained, generally taking the value of 0-4, and a large number of literature studies have taken the value of 2. In this study, "deepSplit=2" is taken as the appropriate value. "cutHeight=0.25" essentially utilizes the characteristic value of the module (representing a certain class of genes). The essence of "cutHeight=0.25" is to construct a tree using the eigenvalues of the modules (representing the expression pattern of a certain type of genes), and then merge the modules belonging to the same branch of the tree and are very close to each other (e.g., Height value ≤ 0.25). The smaller the value, the less easy it is to merge, and the more modules will be retained.

8. **Line 95:** What insights did the Venn analysis provide in terms of shared genes between DM and NPC? How were these shared genes further analyzed using Gene Ontology (GO) and Kyoto Encyclopedia analyses (KEGG)?

**Response:**

As shown in **Line 174 to Line 191**. After GO and KEGG analyses, the red gene module from DM and the purple module from NPC were enriched in the functional pathways of "negative regulation of viral genome replication" and "response to type I interferon", indicating the existence of disease-related shared genes. The red gene module from DM and the purple module from NPC were enriched for "negative regulation of viral genome replication" and "response to type I interferon", indicating the presence of shared disease-related genes. By Venn analysis, 49 shared genes (defined as gene set 1) were obtained from the red gene module of DM origin and the purple module of NPC.

9. **Line 100:** Can you provide more details about the protein-protein interaction (PPI) networks constructed using the STRING tool? How were the parameters "degree cutoff=6", "node score cutoff=0.2", and "K-core=6" determined for the MCODE algorithm?

**Response**:

We are so grateful for your kind question. STRING is one of the most data-rich and widely used databases for studying protein interactions, which contains information on a large number of direct or indirect protein interactions in multiple species. In this study, DM and NPC differential genes were mapped to the online STRING data relative to healthy control samples to obtain the interaction network diagram (PPI) of the corresponding proteins. Each node in the PPI represents a protein, and the lines between the nodes represent the interactions between two proteins, and the more edges there are indicate the existence of multiple interactions between the proteins. The protein networks were clustered to construct functional modules by the GO/KEGG function in the MCODE algorithm ClueGO in Cytoscape software.

MCODE algorithm parameters: 1. Node Score value reflects the denseness of the node and its surrounding nodes, and represents the degree of permissible deviation from the core node. the smaller and stricter the score value is, the smaller the network is obtained. In this study, the parameter "Node Score cutoff=0.2" is often chosen to construct closely connected protein interactions sub-networks. 2. k-core value is one of the commonly used reference values for judging the importance of a node and its sub-networks in terms of the degree of its close connection, and the bigger the k-core value is, the fewer sub-networks will be screened out. In this study, "K-core=6" is used to filter out sub-networks with maximum correlation less than 6 and exclude sub-networks with small score. 3. Degree Cutoff This value controls the minimum degree (number of connections) required to score a node, and the degree of a node in a sub-network needs to be d ≥ the K-core value, thus degree cutoff=6.

10. **Line 107:** How did you decide on the cutoff values "∣logFC∣=1" and "adjusted p-values<0.05" for categorizing genes as up-regulated or down-regulated?

**Response:**

Thank you for your kind question. ∣logFC∣=1 is the differential gene for which the gene expression level of the disease group samples was up- or down-regulated by a factor of 2 compared with that of the healthy control population, and it is also necessary to satisfy the adjusted P-value of <0.05, which indicates that the difference in the expression of the genes is statistically significant.

11. **Line 108:** What were the key biological processes identified by ClueGO based on common DEGs between DM and NPC?

**Response:**

As shown in **Line 197-Line 201**, 144 shared differential genes were identified between DM and NPC, of which 52 differential genes were down-regulated (Figure 5C), and functional enrichment analyses were performed to obtain the key biological processes "negative regulation of viral genome replication" and "establishment of skin barrier" (Figure 5D). In addition, 92 shared differential genes were up-regulated, and the key biological processes enriched were "positive regulation of monocyte chemotaxis" and "microglial cell activation", the results are shown in Fig. S3A-S3B Fig.

12. **Line 125:** Could you provide more details about CIBERSORT and CIBERSORT-R package? How were these tools used to predict immune cell infiltration levels within pathological tissues? Can you add the reference?

**Response:**

Thank you very much for your comments and professional advice. **Line 143**, we have added reference. CIBERSORT is a tool for deconvolution of expression matrices of human immune cell subtypes based on the principle of linear support vector regression. It is used for deconvolution analysis of microarray expression matrices against unknown mixtures and expression matrices containing similar cell types. First, the LM22 data file was downloaded through the CIBERSORT database, which records gene expression characterization data for 22 immune cell types. The expression matrix data were subsequently processed to make them conform to the data files analysed by CIBERSORT. Then, based on the LM22 data, the immune cell infiltration proportion (or immune cell infiltration abundance) in each sample of the dataset could be calculated by the correlation function in the CIBERSORT- R package, and the sum of all immune cell proportions was 1. Whether there was a difference in the level of infiltration of each immune cell between the groups could be determined based on the P-value ＜0.05.

13. **Line 133**: How did you assess the ability of pivotal genes to predict disease using pROC analysis? What were the specific criteria for categorizing genes into two expression level groups?

**Response**:

We thank the reviewer for raising this question. The ROC curve is a tool commonly used to evaluate the performance of classification models, and the accuracy of the model can be determined by comparing the area under the ROC curve (AUC). In this study, we applied the roc function in the pROC-R package to plot the ROC curves of hub genes and calculate the AUC values. The closer the AUC is to 1.0, the higher the performance of genetic diagnosis in predicting related diseases. **Line 231-Line 233**, the AUC values of hub genes'(BST2, IFIT3, IFIH1 and GBP1) were all >0.8, suggesting that hub genes have good disease prediction performance.

According to the median expression level of gene hub genes in the samples, hub genes were categorized into high expression and low expression groups. Functional enrichment of hub genes under high and low expression respectively was explored by GSEA analysis method.

14. **Line 148**: Could you provide more context on the significance of the co-expression modules that showed significant positive correlations with DM and NPC? How do these modules reflect the underlying molecular mechanisms associated with the conditions?

**Response**:

Genes with similar expression patterns or functions were clustered into the same module, and co-expression modules by WGCNA analysis. Correlation analysis (P < 0.05) was performed using these co-expression modules and disease phenotype data, which led to the identification of key gene modules that were significantly associated with disease phenotypes. The importance of these key modules stems from their potential role in disease onset and progression.

Based on gene expression, functional enrichment analysis of genes within these key gene modules can yield molecular functional pathways and biological processes associated with disease development.

15. **Line 157:** How were the shared genes between DM and NPC identified and validated? What were the specific criteria for selecting these shared genes?

**Response:**

As the flow shown in **Fig 1**, in the text reflects that the training set (GSE46239, GSE12452) identified key gene modules significantly associated with DM and NPC phenotypes, respectively, by WGCNA analysis. GO/KEGG enrichment analysis revealed that the key gene modules of DM and NPC directly share the same biological processes and functional pathways "negative regulation of viral genome replication". The key gene modules of both of them shared gene set 1 (gene set 1) after taking the intersection.

In order to validate the results of the training set regarding the existence of a common mechanistic pathway between DM and NPC, we went through the validation set (GSE142807, GSE53819). Differential genes derived from DM and NPC respectively were taken to intersect and 52 shared expression down-regulated differential genes were obtained (gene set 2). Functional enrichment analysis of gene set 2 revealed the same functional pathway enrichment between DM and NPC as the training set. It suggests that there are genes with the same expression pattern and biological function between gene set 1 and gene set 2. The two gene sets were taken to intersect to obtain the shared hub genes of DM and NPC.

The sharing of hub genes between diseases often suggests that this group of genes not only originates from modules that are significantly associated with the disease phenotype, but also that gene expression varies significantly in both DM and NPC. This implies that these genes may play important roles in both diseases. Heatmap and functional enrichment analyses can then provide further insight into the expression patterns and potential biological functions of these genes to determine whether they truly share a common signature. Overall, this process aims to find those genes that play key roles in both DM and NPC, providing clues for further research into the pathogenesis and therapeutic approaches of these two diseases.

16. **Line 161:** Could you elaborate on how the Gene Ontology (GO) analysis and Kyoto Encyclopedia analyses (KEGG) provided insights into the biological processes and pathways related to viral infections and immune responses?

**Response:**

GO is a functional annotation system based on genomic sequence data. It describes the function of genes or proteins by grouping them into different functional categories (e.g., biological processes, cellular components, and molecular functions).

KEGG Analysis is a metabolic pathway database and analysis tool based on genome sequence data. It contains a series of metabolic pathway maps that have been experimentally validated, covering a variety of important biological processes and pathways in organisms. The shared genes of DM and NPC were inputted into the GO or KEGG database, respectively. And the genes or gene products were functionally categorized and annotated according to the annotation information in the database. Whether the biological process or functional pathway was significantly enriched in the sample was judged based on P value < 0.05. **As shown in Fig 3**, 189 shared genes were obtained by taking the intersection in the modules significantly related to DM and NPC respectively. These gene functions were mainly enriched in virus infection-related biological processes and immune response functional pathways. The mechanisms of viral infection and immune response in DM and NPC as well as potential therapeutic targets can be better understood.

17. **Line 166**: How were the protein-protein interaction (PPI) networks constructed for the key modules of DM and NPC? What criteria were used to cluster the PPI network using the MCODE algorithm?

**Response**:

Thank you for your question. Trough the STRING database, a protein interactions network was constructed by inputting genes in the key modules of DM and NPC, respectively. Subsequently, the protein interactions network was clustered into several sub-networks with closer protein interactions by the MCODE algorithm. The criteria for MCODE algorithm to cluster PPI networks were described in question 9.

18. **Line 171:** Can you explain the biological significance of the shared pathways, such as negatively regulating viral genome replication and response to type I interferon, as visualized by ClueGO?

**Response**:

The pathways **“negative regulation of viral genome replication”** means any process that stops, prevents, or reduces the frequency, rate or extent of single stranded viral RNA replication via double stranded DNA intermediate. **“response to type I interferon”** means any process that results in a change in state or activity of a cell or an organism (in terms of movement, secretion, enzyme production, gene expression, etc.) as a result of a type I interferon stimulus. Type I interferons include the interferon-alpha, beta, delta, zeta, kappa, tau, and omega gene families.

19. **Line 179**: What was the rationale for selecting specific cutoff values for categorizing DEGs as up-regulated or down-regulated?

**Response**:

We are so grateful for your kind question. The main reason for choosing specific cutoffs to categorize DEGs (differentially expressed genes) as up- or down-regulated is to clarify which genes are increased and which are decreased in expression. DEG generally means that there is a statistically significant difference in the expressions between two groups of samples. The choice of the specific cutoff value will usually be based on the experimental design and the objectives of the study. Generally, the researcher will determine this value based on statistical significance and biological significance to ensure the reliability and accuracy of the results.

20. **Line 205 and 206**: How do the correlations between immune cell infiltration and hub genes help uncover potential immune mechanisms underlying DM and NPC?

**Response**:

As shown in **Line 221-231**. In DM and NPC, correlation analysis of the pivotal genes with the infiltration level of 22 immune cells revealed that the core genes were significantly positively correlated with the level of M1 infiltration of inflammatory macrophages, and significantly negatively correlated with the level of M2 infiltration of Treg cells in DM and macrophages in NPC. It is suggested that when these hub genes are down-regulated, they may inhibit the involvement of M1 in the immune checking role and increase the involvement of M2 in tumor immune escape, thus promoting tumor growth. In addition, down-regulation of hub genes suppresses immune inflammatory response and may increase the chance of viral infection. Viral infection is not only involved in autoimmune diseases, but also closely related to the development of NPC.

21. **Line 210 and 211:** How were the diagnostic performance and predictive capabilities of hub genes evaluated using the pROC package?

**Response:**

We thank the reviewer for raising this question. The ROC curve is a tool commonly used to evaluate the performance of classification models, and the accuracy of the model can be determined by comparing the area under the ROC curve (AUC). In this study, we applied the roc function in the pROC-R package to plot the ROC curves of hub genes and calculate the AUC values. The closer the AUC is to 1.0, the higher the performance of genetic diagnosis in predicting related diseases. In this study, the AUC values of hub genes'(BST2, IFIT3, IFIH1 and GBP1) were all >0.8, suggesting that hub genes have good disease prediction performance. And they may be valuable biomarkers of DM concurrent NPC.

22. **Line 219:** What insights were gained from the Gene Set Enrichment Analysis (GSEA) regarding the role of hub genes in macrophage-related processes and viral regulation?

**Response:**

The hub genes were categorized into expression up-regulated and down-regulated groups based on the median expression of the hub genes. GSEA gene enrichment analysis was used to obtain the functional enrichment of the core genes when they were up-regulated and down-regulated, respectively. As shown in Fig 10, the core genes were significantly enriched when they were up-regulated to inhibit viral replication, regulate viral processes, positively regulate macrophage factor production and regulate type I interferon-related signaling pathways. The results are similar to the predictions by GeneMANIA database, and further corroborate the previous conclusion that core genes are significantly associated with the level of immune cell infiltration such as macrophages. It suggests that the hub genes are involved in the disease progression of DM and NPC by regulating viral infection and inflammatory responses of immune cells.

23. **Line 235**: Could you explain in more detail how viral infections, such as Coxsackievirus RNA and Coronavirus-19, can trigger or mimic autoimmune diseases like DM? How do these infections influence the immune response and contribute to the pathophysiology of DM?

**Response**:

Thank you for your question. Epstein-Barr virus (EBV) contains encoded proteins that cross-react with host cell antigens, triggering an autoimmune inflammatory response in the host cell, combined with T cell regulatory deficits, leading to autoimmune diseases. EBV also activates human endogenous retroviruses (HERV) ^[24]^. HERV promote pDCs secrete a large amount of IFN α as well as a small fraction of IL-6 and TNF inflammatory factors through the over-activation of pDC cells via the TLR7 receptor. At the same time, the body produces anti-endogenous retroviral envelope glycoprotein gp70 antibody, and the immune-precipitate formed by gp70-anti-gp70 can maintain the activation of pDC to produce IFN α, leading to a vicious circle and aggravating the development of autoimmune diseases^[25]^. Phosphorylated protein 150 of human cytomegalovirus can induces the production of anti-Pp150 autoantibodies. pp150 has a mimetic epitope with similar structure to CIP2A, so the antibody is able to recognize the CD56bright NK cell surface protein CIP2A and induces CD56bright NK cell death through antibody-dependent cytotoxicity (ADCC) and complement-dependent cytotoxicity (CDC) mechanisms, is a common pathological mechanism in patients with a variety of autoimmune diseases^[26]^. Coxsackievirus B causes myositis in patients with Genomic Epigenetic Alterations. Coxsackievirus B induces reduced methylation levels in the promoter region of the skeletal muscle mitochondrial apoptosis activator HRK and promotes HRK expression through activation of the TLR7 receptor, leading to mitochondrial damage and repair dysfunction, resulting in poor myofiber repair, myofiber death, and muscle weakness^[27]^. Coronavirus SARS - CoV - 2 has three immunogenic linear epitope proteins that are highly homologous to dermatomyositis patient sequence, exposure to COVID-19 infection may promote the development of myositis^[28]^. COVID-19 enters host cell through ACE2 receptor and induces the production of myxovirus resistance A in muscle tissue, which promotes the production of I-IFN and leads to muscle damage^[29]^.

24. **Line 263**: Can you elaborate on the potential shared pathophysiology between DM and malignancies, particularly nasopharyngeal carcinoma (NPC)? How might the identified hub genes (IFI44L, IFIT1, IFI6, GBP1, IFX1, OAS1, IFI44, ISG15, IFIT3, and BST2) be involved in this shared pathophysiology?

**Response**:

We appreciate the reviewer for raising this question. As detailed in question 3, Epstein-barr virus infection, down-regulation of type I interferon response, and PD-L1 overexpression reduce immune response and promoting tumour immune escape, which may be a potential common pathological mechanism between dermatomyositis and nasopharyngeal carcinoma. The core genes (IFI44L, IFIT1, IFI6, GBP1, IFX1, OAS1, IFI44, ISG15, IFIT3, and BST2) between DM and NPC were interferon-stimulated genes. As shown in Fig 6, the core gene functions are enriched in regulating viral biological processes and type I interferon signalling pathways. When the negative regulatory viral replication pathway and type I interferon response pathway are down-regulated, not only increases the possibility of viral infection, but also reduces the anti-tumour effect of type I IFN. As shown in Question 3, EBV infection was positively associated with DM complicated with NPC. And EBV can promote viral infection and tumorigenesis by inhibiting type I interferon production and downregulating the RIG - I signalling pathway. Not only is the antiviral effect dependent on type I interferon weakened, but type I interferon-mediated specific T-cell antitumor effects are also attenuated, promoting tumour growth and immune escape (as shown in question 4).

25. **Line 274:** How does miR-146a function as a key immunoregulatory factor in autoimmune diseases like SLE and DM? How does its regulation by type I interferons and Toll-like receptors contribute to its role in these diseases?

**Response:**

Thanks for your kind question. miR-146a is a negative feedback regulator of the natural immune response. miR-146a may play a crucial role in the development of autoimmune diseases. miR-146a down-regulates the expression of interleukin (IL)-1 receptor-associated kinase (IRAK1) and tumour necrosis factor receptor-associated factor 6 (TRAF6) mRNAs by negatively regulating the activation of nuclear factor κ B (NF-κB) mRNA expression. It not only inhibits macrophages produce TNF α, but also regulates the expression of interferon by inhibiting the Toll-like receptor signalling pathway, which helps to control the production of pro-inflammatory cytokines and increase immune tolerance. miR-146a has been reported to be up-regulated in RA, and also elevated in Sjogren's syndrome, and its pathogenesis needs to be further investigated. In contrast, miR-146a expression is defective in PBMCs from SLE patients and is negatively correlated with type I interferon production^[30]^. In addition. miR-146a promotes the maintenance of immune tolerance function of Treg by regulating the increased expression of the target gene STAT1. miR-146a deletion leads to impaired Treg cell function, which in turn led to the disruption of immune tolerance and the development of IFN-γ-mediated immune injury. Conversely, miR-146a deletion and enhanced IFN-γ response may inhibit tumour growth^[31]^. Autoimmune-mediated downregulation of miR-146a expression was found in the lung tissues of patients and mice with diffuse pulmonary hemorrhage, which weakened the negative regulation of TRAF6 and led to increased NETosis formation and apoptosis. Therefore, modulation of miR-146a expression may be a new therapeutic strategy^[32]^.The SLE risk variant rs2431697 regulates the expression of miR-146a and its target genes, which in turn affects the type I interferon pathway to promote SLE pathogenesis. CRISP-based activation of the rs2431697 distal specific enhancer, which binds to the miR-146a promoter and promotes miR-146a expression, attenuates the activation of the type I interferon pathway^[33]^. In SLE patients, high expression of type I IFN inhibits miR-146a maturation by up-regulating monocyte chemotactic protein-inducible protein 1 (MCPIP-1) expression, promoting a vicious cycle of inflammatory response and uncontrolled inflammation. This also partly explains the massive type I IFN expression and its uncontrolled production inducing progressive exacerbation of SLE after exposure to viral infection^[34]^. miR-146a inhibited the migratory capacity of inflammatory macrophages by regulating the expression of macrophage REG3A protein. However, the expression level of miR-146a was significantly lower in PBMCs from PM/DM patients compared to healthy controls, resulting in significantly higher expression of REG3A, IFN-γ and IL-17A, which increased inflammatory macrophage migration^[35]^. It has also been found that serum miR-146a levels are significantly elevated in patients with DM combined with myocarditis compared to DM patients without myocarditis, and further studies are needed to clarify the mechanism of action^[36]^.

A variety of TLRs have been shown to induce IFN - a, IFN - b production. TLR4 expressed on the surface of human cells can be stimulated by LPS and some viral proteins and is an inducer of type I IFN. The pathways by which different Toll-like receptors induce interferon production vary. TLR3 and TLR4 induce IFN - β production through activation of TRIF - IRF3, whereas TLR7, TLR8, and TLR9 induce IFN - α production through activation of MyD88 - IRF7, and IFN - α production through activation of MyD88 - IRF1 or MyD88 - TRAF6-NEMO - TANK - IRF3 pathways. TANK - IRF3 pathway induces IFN - β. Defects in TLRs increase susceptibility to viral infection, and impairment of the TLR3 signalling pathway results in abnormally attenuated production of IFN - α, IFN - β, and IFN - γ, enhanced viral replication, and enhanced fibroblast death. Thus, the TLR-IFN signalling pathway plays an important role for protective immunity against viral infection. A number of studies have shown that viruses have generated multiple strategies to counteract host IFN-mediated antiviral immune responses, but IFN, which can sense viral replication through induced cytoplasmic recognition receptors (e.g., MDA5), may be critical for controlling viral infections^[37]^.COVID - 19 induces IFN - I through the TLR7 / MyD88 axis, whereas IL - 6 production is dependent on TLR2 and TLR2 / 6 perception of SARS - CoV - 2 envelope proteins. SARS - CoV - 2 reduces IFN - I expression by binding to pDC neurociliin-1 reduces TLR7-induced IFN-I expression, an immune evasion strategy of the virus^[38]^. IFN-γ initiates STAT1 signalling via type I IFN or by itself to enhance the macrophage TLR activation response while thereby inducing IL-6/IL-12 family cytokine production to regulate the transition from intrinsic to acquired immunity. Strong activation of TLR4 is associated with Th1 responses, and upon binding of activated TLR2 to the pattern recognition receptor Dectin-1, macrophages produce large amounts of IL-6 and IL-23, which promotes the differentiation of Th17 cells. In addition, TLR2 induced IL-10 expression through the PI3K/Akt/AP-1 pathway, and IL-10 exerted a potent immunosuppressive effect on DCs and macrophages through activation of STAT3 signalling, whereas IFN-γ was able to inhibit TLR2-mediated expression of IL-10 and inhibit STAT3 activation, thereby blocking the feedback inhibitory loop of IL-10 and amplifying inflammatory responses^[39]^. MDA5, encoded by the IFIH1 gene, is a key receptor for viral double-stranded RNA (dsRNA), and the immune complex formed by MDA5 and anti-MDA5 induces type I IFN production through TLR7 in an RNA-dependent manner, providing additional evidence for the involvement of type I IFN in the pathogenesis of anti-MDA5+ DM^[40]^.

26. **Line 297:** Could you provide more insight into how the identified hub genes (IFI44L, IFIT1, IFI6, GBP1, IFX1, OAS1, IFI44, ISG15, IFIT3, and BST2) influence viral infection, immune responses, and potentially the development of malignancy? How do these genes interact with each other and with other cellular processes?

**Response:** Thank you for your questions. The hub genes are interferon-stimulated genes((ISGs)) that have antiviral effector functions^[41]^. As shown in Fig 6, the core genes and their co-expressed genes mainly regulate viral biological processes and type I IFN signalling pathways. As described in question 2, We have elucidated that type I IFN is a very important pathogenetic mechanism in dermatomyositis. Also, type I IFN mediates tumour growth process, drug resistance and radiotherapy resistance (shown in question 4). Down-regulation of core gene expression reduces type I IFN production and promotes viral immune escape. EBV is not only involved in the pathogenesis of autoimmune diseases, but also promotes NPC cells growth. Suppression of type I IFN signalling enhances tumour immune escape, drug resistance, and thus promotes tumour growth. Therefore, the interaction between viral infection and type I IFN response may be an important mechanism for DM complicating NPC.

1. Siming Gao and others, ‘Using Multi-Omics Methods to Understand Dermatomyositis/Polymyositis’, *Autoimmunity Reviews*, 16.10 (2017), 1044–48 <https://doi.org/10.1016/j.autrev.2017.07.021>.
2. Stephanie M Stanford and Nunzio Bottini, ‘PTPN22: The Archetypal Non-HLA Autoimmunity Gene’, 2015.
3. Siming Gao and others, ‘Using Multi-Omics Methods to Understand Dermatomyositis/Polymyositis’, Autoimmunity Reviews, 16.10 (2017), 1044–48 <https://doi.org/10.1016/j.autrev.2017.07.021>.
4. Lu Gan and others, ‘Twins Discordant for Myositis and Systemic Lupus Erythematosus Show Markedly Enriched Autoantibodies in the Affected Twin Supporting Environmental Influences in Pathogenesis’, *BMC Musculoskeletal Disorders*, 15.1 (2014), 67 <https://doi.org/10.1186/1471-2474-15-67>.
5. Akinori Uruha and others, ‘Sarcoplasmic MxA Expression: A Valuable Marker of Dermatomyositis’, *Neurology*, 88.5 (2017), 493–500 <https://doi.org/10.1212/WNL.0000000000003568>.
6. C. Thompson, V. Piguet, and E. Choy, ‘The Pathogenesis of Dermatomyositis’, *British Journal of Dermatology*, 179.6 (2018), 1256–62 <https://doi.org/10.1111/bjd.15607>.
7. Gulnara Mamyrova and others, ‘Environmental Factors Associated with Disease Flare in Juvenile and Adult Dermatomyositis’, *Rheumatology*, 56.8 (2017), 1342–47 <https://doi.org/10.1093/rheumatology/kex162>.
8. Meredyth G Ll Wilkinson and others, ‘Role of CD14+ Monocyte-Derived Oxidised Mitochondrial DNA in the Inflammatory Interferon Type 1 Signature in Juvenile Dermatomyositis’, *Annals of the Rheumatic Diseases*, 82.5 (2023), 658–69 <https://doi.org/10.1136/ard-2022-223469>.
9. Xin Lu and others, ‘The Host Defense Peptide LL-37 a Possible Inducer of the Type I Interferon System in Patients with Polymyositis and Dermatomyositis’, *Journal of Autoimmunity*, 78 (2017), 46–56 <https://doi.org/10.1016/j.jaut.2016.12.003>.
10. M. Gasparotto and others, ‘The Interferon in Idiopathic Inflammatory Myopathies: Different Signatures and New Therapeutic Perspectives. A Literature Review’, *Autoimmunity Reviews*, 22.6 (2023), 103334 <https://doi.org/10.1016/j.autrev.2023.103334>.
11. Gallay Laure and others. ‘Involvement of Type I Interferon Signaling in Muscle Stem Cell Proliferation During Dermatomyositis’, *Neurology* vol. 98,21 (2022): e2108-e2119. <https://doi:10.1212/WNL.0000000000200271>.
12. Pei-Yu Huang and others, ‘Paired Study of 172 Cases of Nasopharyngeal Carcinoma with or without Dermatomyositis’, *Acta Oto-Laryngologica*, 134.8 (2014), 824–30 <https://doi.org/10.3109/00016489.2014.913312>.
13. Der-Yuan Chen and others, ‘Polymyositis/Dermatomyositis and Nasopharyngeal Carcinoma: The Epstein–Barr Virus Connection?’, *Journal of Clinical Virology*, 49.4 (2010), 290–95 <https://doi.org/10.1016/j.jcv.2010.08.015>.
14. Chongfeng Xu and others, ‘Latent Membrane Protein 1 of Epstein–Barr Virus Promotes RIG-I Degradation Mediated by Proteasome Pathway’, *Frontiers in Immunology*, 9 (2018), 1446 <https://doi.org/10.3389/fimmu.2018.01446>.
15. Noppadol Larbcharoensub and others, ‘Characterization of PD-L1 and PD-1 Expression and CD8+ Tumor-Infiltrating Lymphocyte in Epstein-Barr Virus-Associated Nasopharyngeal Carcinoma’, *American Journal of Clinical Oncology*, 41.12 (2018), 1204–10 <https://doi.org/10.1097/COC.0000000000000449>.
16. He Chen and others, ‘Increased Levels of Soluble Programmed Death Ligand 1 Associate with Malignancy in Patients with Dermatomyositis’, *The Journal of Rheumatology*, 45.6 (2018), 835–40 <https://doi.org/10.3899/jrheum.170544>.
17. Samuel Knauss and others, ‘PD1 Pathway in Immune-Mediated Myopathies: Pathogenesis of Dysfunctional T Cells Revisited’, *Neurology - Neuroimmunology Neuroinflammation*, 6.3 (2019), e558 <https://doi.org/10.1212/NXI.0000000000000558>.
18. Jung-Lung Hsu and others, ‘Reappraisal of the Incidence, Various Types and Risk Factors of Malignancies in Patients with Dermatomyositis and Polymyositis in Taiwan’, *Scientific Reports*, 11.1 (2021), 4545 <https://doi.org/10.1038/s41598-021-83729-5>.
19. Y.L. Huang and others, ‘Malignancies Associated with Dermatomyositis and Polymyositis in Taiwan: A Nationwide Population-Based Study’, *British Journal of Dermatology*, 161.4 (2009), 854–60 <https://doi.org/10.1111/j.1365-2133.2009.09274.x>.
20. T H Tai, ‘PETER TEO, MBBS(HK), FRCR(UK), DMRT(LoND),’ DAMON CHOY, MBBS(HK), FRCR(UK),’.
21. Liufu Deng and others, ‘STING-Dependent Cytosolic DNA Sensing Promotes Radiation-Induced Type I Interferon-Dependent Antitumor Immunity in Immunogenic Tumors’, *Immunity*, 41.5 (2014), 843–52 <https://doi.org/10.1016/j.immuni.2014.10.019>.
22. Zikun Ma and others, ‘AhR Diminishes the Efficacy of Chemotherapy via Suppressing STING Dependent Type-I Interferon in Bladder Cancer’, *Nature Communications*, 14.1 (2023), 5415 <https://doi.org/10.1038/s41467-023-41218-5>.
23. Jun-Yan Li and others, ‘TRIM21 Inhibits Irradiation-Induced Mitochondrial DNA Release and Impairs Antitumour Immunity in Nasopharyngeal Carcinoma Tumour Models’, *Nature Communications*, 14.1 (2023), 865 <https://doi.org/10.1038/s41467-023-36523-y>.
24. David H. Dreyfus, ‘Autoimmune Disease: A Role for New Anti-Viral Therapies?’, *Autoimmunity Reviews*, 11.2 (2011), 88–97 <https://doi.org/10.1016/j.autrev.2011.08.005>.
25. Lucie Baudino and others, ‘Role of Endogenous Retroviruses in Murine SLE’, *Autoimmunity Reviews*, 10.1 (2010), 27–34 <https://doi.org/10.1016/j.autrev.2010.07.012>.
26. Yu Liu and others, ‘A Cytomegalovirus Peptide-Specific Antibody Alters Natural Killer Cell Homeostasis and Is Shared in Several Autoimmune Diseases’, *Cell Host & Microbe*, 19.3 (2016), 400–408 <https://doi.org/10.1016/j.chom.2016.02.005>.
27. Jessica F Boehler and others, ‘Mitochondrial Dysfunction and Role of Harakiri in the Pathogenesis of Myositis’, *The Journal of Pathology*, 249.2 (2019), 215–26 <https://doi.org/10.1002/path.5309>.
28. Spyridon Megremis and others, ‘Antibodies against Immunogenic Epitopes with High Sequence Identity to SARS-CoV-2 in Patients with Autoimmune Dermatomyositis’, *Annals of the Rheumatic Diseases*, 79.10 (2020), 1383–86 <https://doi.org/10.1136/annrheumdis-2020-217522>.
29. Jie Qian and Hui Xu, ‘COVID-19 Disease and Dermatomyositis: A Mini-Review’, *Frontiers in Immunology*, 12 (2022), 747116 <https://doi.org/10.3389/fimmu.2021.747116>.
30. Edward K L Chan, Angela Ceribelli, and Minoru Satoh, ‘MicroRNA-146a in Autoimmunity and Innate Immune Responses’, *Annals of the Rheumatic Diseases*, 72.suppl 2 (2013), ii90–95 <https://doi.org/10.1136/annrheumdis-2012-202203>.
31. Stefan Emming, Michele Chirichella, and Silvia Monticelli, ‘MicroRNAs as Modulators of T Cell Functions in Cancer’, *Cancer Letters*, 430 (2018), 172–78 <https://doi.org/10.1016/j.canlet.2018.05.019>.
32. Yu-Tung Hsieh and others, ‘Down-Regulated miR-146a Expression with Increased Neutrophil Extracellular Traps and Apoptosis Formation in Autoimmune-Mediated Diffuse Alveolar Hemorrhage’, *Journal of Biomedical Science*, 29.1 (2022), 62 <https://doi.org/10.1186/s12929-022-00849-4>.
33. Guojun Hou and others, ‘SLE Non-Coding Genetic Risk Variant Determines the Epigenetic Dysfunction of an Immune Cell Specific Enhancer That Controls Disease-Critical microRNA Expression’, *Nature Communications*, 12.1 (2021), 135 <https://doi.org/10.1038/s41467-020-20460-1>.
34. Bo Qu and others, ‘Type I Interferon Inhibition of MicroRNA‐146a Maturation Through Up‐Regulation of Monocyte Chemotactic Protein–Induced Protein 1 in Systemic Lupus Erythematosus’, *Arthritis & Rheumatology*, 67.12 (2015), 3209–18 <https://doi.org/10.1002/art.39398>.
35. Tingwang Jiang and others, ‘Reduced miR-146a Promotes REG3A Expression and Macrophage Migration in Polymyositis and Dermatomyositis’, *Frontiers in Immunology*, 11 (2020), 37 <https://doi.org/10.3389/fimmu.2020.00037>.
36. Yue Zhang and others, ‘Identification of Key Biomarkers Associated with Immune Cells Infiltration for Myocardial Injury in Dermatomyositis by Integrated Bioinformatics Analysis’, *Arthritis Research & Therapy*, 25.1 (2023), 69 <https://doi.org/10.1186/s13075-023-03052-4>.
37. Shen-Ying Zhang and others, ‘Human Toll-like Receptor-Dependent Induction of Interferons in Protective Immunity to Viruses’, *Immunological Reviews*.
38. Jennifer Becker and Ulrich Kalinke, ‘Toll‐like Receptors Matter: Plasmacytoid Dendritic Cells in COVID‐19’, *The EMBO Journal*, 41.10 (2022), e111208 <https://doi.org/10.15252/embj.2022111208>.
39. Xiaoyu Hu, Soumya D Chakravarty, and Lionel B Ivashkiv, ‘Regulation of Interferon and Toll-like Receptor Signaling during Macrophage Activation by Opposing Feedforward and Feedback Inhibition Mechanisms’, *Immunological Reviews*, 2008.
40. Kaiwen Wang and others, ‘RNA-Containing Immune Complexes Formed by Anti-Melanoma Differentiation Associated Gene 5 Autoantibody Are Potent Inducers of IFN-α’, *Frontiers in Immunology*, 12 (2021), 743704 <https://doi.org/10.3389/fimmu.2021.743704>.
41. John W Schoggins and Charles M Rice, ‘Interferon-Stimulated Genes and Their Antiviral Effector Functions’, *Current Opinion in Virology*, 1.6 (2011), 519–25 <https://doi.org/10.1016/j.coviro.2011.10.008>.
